# Relationship between risk information on total colonoscopy and patient preferences for colorectal cancer screening options: Analysis using the Analytic Hierarchy Process

**DOI:** 10.1186/1472-6963-8-106

**Published:** 2008-05-21

**Authors:** Yuichi Katsumura, Hideo Yasunaga, Tomoaki Imamura, Kazuhiko Ohe, Hiroshi Oyama

**Affiliations:** 1 Department of Health Informatics, Graduate School of Medicine, University of Tokyo, Tokyo, Japan; 2 Department of Health Management and Policy, Graduate School of Medicine, University of Tokyo, Tokyo, Japan; 3 Department of Public Health, Health Management and Policy, Nara Medical University, Nara, Japan; 4 Department of Medical Informatics and Economics, Graduate School of Medicine, University of Tokyo, Tokyo, Japan; 5 Department of Clinical Information Engineering, School of Public Health, University of Tokyo, Tokyo, Japan

## Abstract

**Background:**

Although the fecal occult blood test (FOBT) is the preferred program for colorectal cancer screening in Japan, many medical institutions have recently begun to provide total colonoscopy (TCS) as an initial screening program. However, there are a number of severe risks associated with TCS, such as colorectal bleeding and perforation. The justification for performing such a procedure on healthy patients remains unclear. We used the analytic hierarchy process (AHP) to investigate whether risk information on TCS affects patient preferences for colorectal cancer screening.

**Methods:**

We performed a questionnaire survey using an AHP decision-making model, targeting 285 people aged 40–59 years. We randomly assigned the subjects into Groups A (n = 146) and B (n = 139). Both groups were provided with information on the effectiveness, cost and disadvantages of the two screening programs. Group A was provided with additional information regarding the risks of TCS. Individual priorities were calculated with pair-wise comparisons between the two alternatives in each selection criteria. The influence of the risk information was analyzed using a logistic regression analysis.

**Results:**

The aggregated priorities in Group A for 'effectiveness', 'costs', and 'risks' were 0.603, 0.147, and 0.250, respectively, while those in Group B were 0.652, 0.149, and 0.199, respectively. A logistic regression analysis showed that the provision of risk information significantly reduced the subjects' priorities for TCS (p = 0.036).

**Conclusion:**

The lack of risk information was related to the differences in priorities assigned to effectiveness and risks of the two procedures. Patients must be well informed before making decisions concerning their preferred colorectal cancer screening procedure.

## Background

In Japan, colorectal cancer is on the increase as a cause of death [1]. Cancer screening is considered one of the most promising approaches in the prevention of cancer deaths.

The fecal occult blood test (FOBT) is the preferred program for colorectal cancer screening in Japan, being a non-invasive, low-cost procedure. Subjects with positive FOBT results are recommended to undergo total colonoscopy (TCS), which is relatively invasive and costly; however, FOBT has a number of limitations, including false negative and false positive results. Many hospitals in Japan provide TCS to healthy individuals as an initial screening program; however, there are a number of severe risks associated with TCS, such as colorectal bleeding and perforation. The justification for performing such a procedure on healthy patients remains unclear.

It is necessary to provide the general population with appropriate information on cancer screening. The information should focus on the risks as well as the effectiveness and costs associated with various procedures. People can then select their preferred service on the basis of all the necessary information.

Although previous studies have examined patient preferences concerning colorectal cancer screening [2-6], there is little agreement as to which test is more preferred. Pignone et al. reported that patient preferences for colorectal cancer screening were sensitive to information regarding test performance and cost information [6]; however, to our knowledge, the actual impact of risk information on patient preferences remains unknown.

In the present study, we used the analytic hierarchy process (AHP) to analyse peoples' preferences for colorectal cancer screening. AHP is a flexible decision-making method developed by Saaty in the 1970s [7] to help people establish priorities and make the best decision, when both the qualitative and quantitative aspects of a decision need to be considered.

With regard to healthcare services, it appears to be somewhat difficult for physicians to communicate fair and sufficient information to patients due to physician-specific biases [8], limited diagnosis and treatment time [9], and the limitations of physicians' interviewing skills [10].

AHP not only helps patients arrive at the best decision;, it also provides a clear rationale by reducing complex decisions to a series of one-on-one comparisons, thereafter synthesizing the results. In the AHP model, the alternative selection problem is structured into a hierarchy, with the overall goal placed at the top, with lower-level selection criteria below. Alternatives are then assigned weights at each level of the hierarchy, and overall global weights and priorities are obtained.

Several studies have considered the application of AHP in medical decision-making [2,11-13]. The present study aimed to use the AHP method to investigate whether risk information affects people's preferences for colorectal cancer screening procedures.

## Methods

### Construction of the AHP model

**Figure 1:**
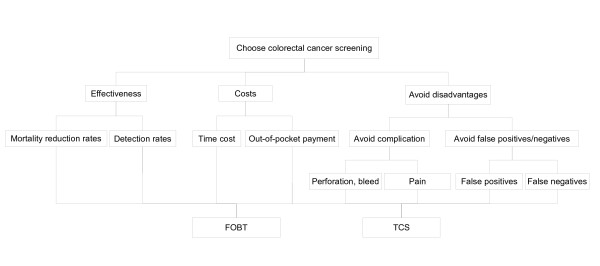
The decision-making model.

From the patient point of view, we constructed an AHP decision model that helps people to make a dichotomous choice between FOBT and TCS as an initial colorectal screening program. We decomposed the decision problem into a hierarchy of more easily comprehended sub-problems (Fig. 1). We focused on the following three criteria related to decision-making in colorectal cancer screening: 'effectiveness', 'costs', and 'disadvantages.'

The 'effectiveness' of the service is further divided into two sub-criteria: 'mortality reduction rate' and 'cancer detection rate'.

The 'costs' are separated into two sub-criteria: 'out-of-pocket payment' and 'time cost'. 'Time cost' represents the indirect cost of the examination time at the expense of working hours.

'Disadvantages' include 'false positives/negatives' and 'risks'. A false positive result can cause anxiety for the subject, whereas a false negative result can lead to delays in diagnosis and treatment. The FOBT presents almost no risk, while TCS can lead to complications such as bleeding and perforation.

### Subjects

This research was based on an Internet-based self-administered questionnaire. We enlisted the services of an Internet research company with approximately 261,000 registered Internet users. We chose 640 males and females via stratified random sampling from a total of approximately 79,000 persons aged 40–59 years.

The cover letter of the questionnaire clearly informed the subjects that 1) data collection and analysis was completely anonymous, ensuring that their private details would remain confidential; 2) all the answers would be kept confidential, processed statistically, and used only for scientific study; and 3) they had a choice whether to participate or not.

Our offer to the subjects to participate in our study was sent by email on 19 December 2006. We obtained replies from 353 people within one week: a response rate of 55.2%.

### Questionnaire

We designed the FOBT and TCS information based on the following evidence-based objective facts. In the present study, FOBT means immunohistochemical test rather than guaiac test.

#### (i) Mortality reduction rates

While several randomized controlled trials have evaluated the mortality reduction rates of FOBT in Western nations, there have been none in Japan. Data from case-control studies are available for Japanese people: Saito et al. reported that the estimated relative risk of mortality for the screened population was 0.48 for FOBT [14].

To our knowledge, no data about mortality reduction rates of TCS are available for Japanese people. Instead, we referred to a foreign data based on a case-control study: the estimated relative risk of mortality for the screened population was 0.41 for TCS [15].

#### (ii) Cancer detection rates

In Japan, the cancer detection rate of FOBT is reportedly 27 per 10,000 persons, while that of TCS is 61 per 10,000 [16].

#### (iii) Out-of-pocket payment

The actual price of FOBT is less than ¥1,000 (about US$9) or free, while that of TCS is ¥15,000–25,000 (US$130–220).(unpublished data)

#### (iv) Time spent for TCS

Generally, in TCS, the use of a laxative in pre-treatment requires approximately 2 hours; the actual examination takes 10–15 minutes. Most colonoscopies in Japan are done without sedation. In the present study, travel or waiting time was not presented.

#### (v) False negatives/false positives

According to several studies, false negative rates are 7–37% and 2–5% in FOBT and TCS, respectively. False positive rates in FOBT are 2–30%, while not reported in TCS [17-21].

#### (vi) Complications

The risk of severe complications with TCS, including perforation and bleeding, has been reported to be approximately 8 per 10,000 colonoscopies without biopsy or polypectomy [22]; FOBT poses almost no risk to the patient.

The additional file 1 shows the "Information Sheet (originally in Japanese)" which was actually presented to the participants. Two types of Information Sheets were prepared, Sheets A and B. Sheet A included items (i)-(vi) above, whereas Sheet B referred only to items (i)-(v), excluding the risk information.

Sheet A was presented randomly to 176 respondents (Group A) and Sheet B to 177 respondents (Group B). Participants then answered a questionnaire based on the AHP model, selecting one item from seven grades for a pair-wise comparison of the factors and alternatives. That is, for their judgement of relative importance between X and Y variables, subjects were asked the following questions: "Which do you think is important, X or Y?" The detailed illustrations with elements substituted for the X and Y variables were as follows:

Q1. Which do you think is important, 'effectiveness' or 'costs' from the point of choosing colorectal cancer screening?

Q2. Which do you think is important, 'effectiveness' or 'avoiding disadvantages' from the point of choosing colorectal cancer screening?

Q3. Which do you think is important, 'costs' or 'avoiding disadvantages' from the point of choosing colorectal cancer screening?

Q4. Which do you think is important, 'mortality reduction' or 'detection rate' from the point of effectiveness?

Q5. Which according to you is important 'co-payment' or 'time cost' from the point of costs?

Q6. Which do you think is important 'complications' or 'false-positives/false-negatives' from the point of avoiding disadvantages?

Q7. Which do you think is important 'false-negatives' or 'false-positives' from the point of avoiding false-positives/negatives?

The participants were requested to choose one answer to each question from the following seven options:

1 X is extremely important.

2 X is strongly important.

3 X is moderately important.

4 Indifferent.

5 Y is moderately important.

6 Y is strongly important.

7 Y is extremely important.

Questions were also included on age, gender, concern about their own health (3 grades: concerned a lot, concerned a little, and not concerned), household annual income, history of hospital admission and personal experience of FOBT and TCS.

### Analysis

A consistency index (C.I.) was calculated for each respondent, reflecting the consistency of qualitative judgments of the importance of different criteria and the impact of the importance on all comparisons [23]. If the C.I. was less than 0.15, we assumed the comparison is consistent [24]. In the present study, respondents with a C.I. greater than or equal to 0.15 were excluded.

Individual priorities were calculated via pair-wise comparisons between the two alternatives in each selection criteria. In the present study, we used geometric mean method (GMM) to estimate individuals' relative weights of each element. Aggregated weights of individual priorities were calculated with GMM for synthesizing individual decisions into a group decision [9]. To derive the group weights for each element, we calculated geometric mean from each individual pair-wise comparison matrix. For all these calculations, we used a programmed spreadsheet established with Microsoft Excel™ software.

We analysed the influence of the risk information on the subjects' decisions using a logistic regression analysis; the results of the dichotomous choice between FOBT and TCS were regressed against a variety of independent variables including the type of information presented and the subjects' backgrounds.

We used the statistical software JMP version 6.0.3 (SAS Institute Inc., Cary, NC, USA) for all statistical analyses. A p value less than 0.05 was considered to be statistically significant.

## Results

### Baseline characteristics

Sixty-eight respondents with a C.I. greater than or equal to 0.15 were excluded, leaving a total of 285 respondents to be analysed. The proportion of inconsistent respondents was 19% (= 68/353). The participants' characteristics of both consistent and inconsistent subjects are shown in Table 1. In the consistent subjects, the average (± SD) of consistency index values were 0.058 ± 0.052 in Group A (n = 146) and 0.063 ± 0.051 in Group B (n = 139).

**Table 1:** Sample Characteristics (n = 353)

Parameters	Included subjects (n = 285)		Excluded subjects (n = 68)	
	
	Group A (n = 146)	Group B (n = 139)	Group A (n = 30)	Group B (n = 38)
Sex				
Male	71 (49%)	63 (45%)	16 (53%)	23 (61%)
Female	75 (51%)	76 (55%)	14 (47%)	15 (39%)
Age (average ± SD)	48.6 ± 6.0	48.7 ± 5.4	50.1 ± 5.8	48.2 ± 5.3
Concerns about own health				
Concerned a lot	58 (40%)	46 (33%)	10 (33%)	11 (29%)
Concerned a little	50 (34%)	53 (38%)	12 (40%)	19 (50%)
Not concerned at all	38 (26%)	40 (29%)	8 (27%)	8 (21%)
Annual household income				
Low (<US$59,999)	35 (24%)	46 (33%)	12 (40%)	14 (37%)
Medium (US$60,000 – 119,999)	76 (52%)	58 (42%)	13 (43%)	17 (45%)
High (>US$120,000)	14 (10%)	11 (8%)	3 (10%)	3 (8%)
No answer given	21 (14%)	24 (17%)	2 (7%)	4 (11%)
Experience of hospital admission				
None	52 (36%)	54 (39%)	16 (53%)	10 (26%)
1 admission	43 (29%)	31 (22%)	5 (17%)	9 (24%)
2 admissions	22 (15%)	31 (22%)	6 (20%)	10 (26%)
3 or more admissions	26 (18%)	22 (16%)	3 (10%)	8 (21%)
No answer given	3 (2%)	1 (1%)	0 (0%)	1 (3%)
Experience of FOBT: Yes	83 (57%)	81 (58%)	20 (67%)	23 (61%)
Experience of TCS: Yes	24 (16%)	30 (22%)	8 (27%)	5 (13%)

### Aggregated weights and priorities

Table 2 shows the global and local priorities and weights of each subcriteria, as well as overall priorities. The aggregated priorities in Group A for 'effectiveness', 'costs', and 'avoiding disadvantages' were 0.603, 0.147 and 0.250, respectively, while those in Group B were 0.652, 0.149, and 0.199, respectively. That is, Group A represented a higher priority to 'avoiding disadvantage' and a lower priority to 'effectiveness' compared to Group B. Overall priorities of TCS were 0.620 and 0.652 in Group A and Group B, respectively.

**Table 2:** Results of comparison between FOBT and TCS (aggregated weights and priorities)

Criterion	Group A				Group B			
	
	Global priority	Local priority	FOBT weight	TCS weight	Global priority	Local priority	FOBT weight	TCS weight
Major criteria								
Effectiveness		0.603	0.220	0.780		0.652	0.202	0.798
Costs		0.147	0.830	0.170		0.149	0.839	0.161
Avoid disadvantages		0.250	0.501	0.499		0.199	0.455	0.545
Subcriteria of 'effectiveness'								
Mortality reduction rates	0.393	0.652	0.237	0.763	0.471	0.723	0.225	0.775
Detection rates	0.210	0.348	0.187	0.813	0.181	0.277	0.192	0.808
Subcriteria of 'costs'								
Time cost	0.071	0.482	0.828	0.172	0.071	0.479	0.817	0.183
Out-of-pocket payment	0.076	0.518	0.832	0.168	0.078	0.521	0.832	0.168
Subcriteria of 'avoid disadvantages'								
Complications	0.163	0.650	0.670	0.330	0.092	0.461	0.790	0.210
False positives/negatives	0.088	0.250	0.185	0.815	0.107	0.539	0.168	0.832
Subcriteria of 'avoid false positives/negatives'								
False positives	0.062	0.706	0.186	0.814	0.076	0.711	0.180	0.820
False negatives	0.026	0.294	0.182	0.818	0.031	0.289	0.177	0.823

Global priority of each subcriterion = local priority of each subcriterion * priority of the parent criterion. e.g., global priority of mortality reduction rates in Group A = 0.652*0.603 = 0.393.

Weight of the parent criterion is the sum of (weight * local priority) of each subcriterion. e.g., FOBT weight of effectiveness in Group A = 0.237*0.652+0.187*0.348 = 0.220.

Overall priority is the sum of (weight * local priority) of each major criterion.

Overall priority of FOBT in Group A = 0.220*0.603+0.830*0.147+0.501*0.250 = 0.380.

Overall priority of TCS in Group A = 0.780*0.603+0.170*0.147+0.499*0.250= 0.620.

Overall priority of FOBT in Group B = 0.202*0.652+0.839*0.149+0.455*0.199= 0.348.

Overall priority of TCS in Group B = 0.798*0.652+0.161*0.149+0.545*0.199 = 0.652.

### Individual weights and priorities

In Group B, 124 (89.2%) of the 146 respondents gave a higher priority to TCS than to FOBT; in Group A, 118 (80.8%)/139 preferred TCS to FOBT. These two figures differ significantly. (p < 0.01)

In the logistic regression analysis, 'type of information' was a significant factor affecting choice between FOBT and TCS (Table 3): those presented with the risk information concerning TCS were more likely to choose FOBT.

**Table 3:** Logistic regression analysis (1: FOBT, 0: TCS)

Independent variables	Odds ratio	95% confidence interval	P value
Group (A: 1, B: 0)	0.443	0.200–0.931	0.036
Sex (male: 1, female: 0)	1.398	0.681–2.915	0.364
Age	1.018	0.301–3.405	0.977
Concerns about own health	1.438	0.347–5.582	0.605
Annual household income	0.693	0.141–3.377	0.649
Experience of hospital admission	2.073	0.786–5.485	0.139
Experience of FOBT (Yes: 1, No: 0)	2.202	1.001–5.041	0.054
Experience of TCS (Yes: 1, No: 0)	1.370	0.427–5.308	0.616

R^2 ^= 0.690

## Discussion

### People's preferences for colorectal cancer screening

Cancer screening exists to save lives. In order to reduce overall morbidity and mortality of cancer, it would be important to increase the participation rate of screening program. Several previous studies indicated that use of "decision-aid" significantly increased the participation rate of colorectal cancer screening [26,27]. AHP can be utilized as a tool for decision aid.

In the present study, we found that fewer subjects expressed a preference for FOBT than for TCS. The subjects placed the greatest weighting on 'effectiveness'. This result indicates that their greatest concern about cancer screening is the life-saving effect.

The relative weight of avoiding the risks of TCS was much smaller than that of its effectiveness. In other words, many people seemed to consider the risks associated with TCS to be small, and did not attach much importance to them: instead, subjects focused on the life-saving aspect of TCS.

Subjects had relatively little concern about the costs of screening. Possibly, the costs of TCS were inconsiderable and affordable to them.

### Necessity of informed consent

Priority of 'effectiveness' was higher in Group B (0.652) than in Group A (0.603), while priority of 'avoid disadvantage' was lower in Group B (0.250) than in Group A (0.199). That is, lack of risk information regarding perforation and bleeding increased weight of 'effectiveness' and decreased weight of 'avoid disadvantages'.

It is necessary to provide the general population with sufficient information on colorectal cancer screening. Such information should include not only the effectiveness but also the risks of screening.

Davey et al. reported that most women wanted information about the possibility of false results and side effects; although such information would make them anxious, they wanted it anyway [25]. However, it is thought that many healthcare professionals and the media tend only to focus on the effectiveness of the screening. Ideally, people should be well informed before making decisions affecting their health; unfortunately, most are ill-informed with regard to screening.

In the present study, the provision of the risk information changed the preferred screening method for 8.4% of the subjects. The priority for TCS in Group B can be regarded as an overestimation due to a lack of information. However, >80% of the subjects in Group A still preferred TCS in spite of the provision of risk information. These results suggest that many people can balance the concerns of disadvantages against the effectiveness of saving lives.

### Limitations of the present study

In the present study, several limitations should be acknowledged.

First, we chose our subjects from Internet users in Japan. Although approximately 80% of Japanese homes now have access to the Internet, Internet users as a group may be biased toward those with higher levels of education and higher annual incomes. The second limitation is the self-selection bias. Third, the model we used did not consider effect of polyp detection. One of the objectives of colorectal cancer screening programs is to identify and remove potentially pre-cancerous polyps. Fourth, several data presented were only estimates with broad ranges. It is possible that use of different estimates could have affected the study results. Fifth, the subjects' reactions to the information, especially disadvantages, could have been affected by the way the information was formatted. Sixth, excluding the inconsistent subjects could be a limitation because of unequal characteristics between the consistent and inconsistent groups. Although statistically insignificant, the male ratio was substantially high in the inconsistent group. Seventh, the local priorities of the 'effectiveness' sub-criterion differed between Group A and B, although they should be similar because there was no difference in the information presented. This disparity may have been caused by the inequality of the distribution of participants' characteristics, particularly the difference of concerns about their own health.

## Conclusion

In the present study, we investigated whether risk information affects people's preferences to colorectal cancer screening using AHP. The results suggest that there is a relationship between the lack of risk information and the differences in priorities assigned to effectiveness and risks of the two procedures. Patients should be well informed of both the risks and benefits before making decisions on selecting colorectal cancer screening options.

## Competing interests

The authors declare that they have no competing interests.

## Authors' contributions

All authors jointly conceived of the idea for this study. YK and HY constructed the model, and TI, HO and KO validated the content. YK analysed the data, and all authors interpreted the results. YK and HY drafted the manuscript. All authors revised the paper and approved the final version. All authors take public responsibility for the content of the manuscript.

## Pre-publication history

The pre-publication history for this paper can be accessed here:



## Supplementary Material

Additional file 1 Information sheets (Originally in Japanese). Comparison of fecal occult blood test (FOBT) and total colonoscopy (TCS). Click here for file
