# Supplementary material for: Relationship between risk information on total colonoscopy and patient preferences for colorectal cancer screening options: Analysis using the Analytic Hierarchy Process
Source: BMC Health Serv Res. 2008 May 21;8:106. doi: 10.1186/1472-6963-8-106 (PMC2412845; doi:10.1186/1472-6963-8-106)
Supplement: Additional file 1 — Information sheets (Originally in Japanese). Comparison of fecal occult blood test (FOBT) and total colonoscopy (TCS). [file 1472-6963-8-106-S1.doc]

**Additional file 1. Information sheets (Originally in Japanese)**

For colon cancer screening, two different options are available: fecal occult blood test (FOBT) and total colonoscopy (TCS). These two tests differ in ‘effectiveness’, ‘costs’ and ‘disadvantages’.

#1 Effectiveness

(i) Mortality reduction rates

Colon cancer screening is expected to reduce the risk of dying from colon cancer. Studies have demonstrated that FOBT reduces the death rate by ~52%, while TCS by ~59%.

(ii) Cancer detection rates

The cancer detection rate of FOBT is reportedly 27 per 10,000 persons, while that of TCS is 61 per 10,000.

#2 Costs

(iii) Out-of-pocket payment

The actual price of FOBT is <¥1,000 or free, while that of TCS is ¥15,000–25,000.

(iv) Time cost for TCS

Generally, in TCS, the use of a laxative in pre-treatment requires ~2 h; the actual examination takes 10–15 min.

#3 Disadvantages

(v) False-negatives/false-positives

The presence of cancer cannot be ruled out 100%, even if the screening tests are negative. Studies show that false-negatives occur at a rate of 7–37% and 2–5% in FOBT and TCS, respectively.

Positive screening results do not necessarily mean actual presence of cancer. Studies show that false-positive rates in FOBT are 2–30%. False-positives in TCS have not been statistically reported; they are considered negligible.

(vi) Complications

The risk of severe complications with TCS, including perforation and bleeding, has been reported to be ~8 per 10,000 TCS. FOBT poses almost no risk.
